# Ultrashort large-bandwidth X-ray free-electron laser generation with a dielectric-lined waveguide

**DOI:** 10.1107/S1600577524000249

**Published:** 2024-02-09

**Authors:** Yiwen Liu, Zhen Wang, Lingjun Tu, Chao Feng, Zhentang Zhao

**Affiliations:** a School of Physical Science and Technology, ShanghaiTech University, Shanghai 201210, China; b Shanghai Institute of Applied Physics, Chinese Academy of Sciences, Shanghai 201800, China; c Shanghai Advanced Research Institute, Chinese Academy of Sciences, Shanghai 201210, China; d Institute of Advanced Science Facilities, Shenzhen 518107, China; e University of Chinese Academy of Sciences, Beijing 100049, China; RIKEN SPring-8 Center, Japan

**Keywords:** free-electron laser, dielectric wakefield, large-bandwidth X-ray, terahertz

## Abstract

Large-bandwidth pulses produced by cutting-edge X-ray free-electron lasers (FELs) are of great importance in research fields like material science and biology. In this paper, a new method to generate high-power ultrashort FEL pulses with tunable spectral bandwidth with spectral coherence using a dielectric-lined waveguide without interfering operation of linacs is proposed. By exploiting the passive and dephasingless wakefield at terahertz frequency excited by the beam, stable energy modulation can be achieved in the electron beam and large-bandwidth high-intensity soft X-ray radiation can be generated. Three-dimensional start-to-end simulations have been carried out and the results show that coherent radiation pulses with duration of a few femtoseconds and bandwidths ranging from 1.01% to 2.16% can be achieved by changing the undulator taper profile.

## Abstract 2

Ultrashort large-bandwidth X-ray free-electron laser generation is proposed using a dielectric-lined waveguide. With this modulation scheme through strong terahertz wakefield self-excited, a cheap and flexible large-bandwidth operation mode is researched for free-electron-laser complexes.

## 1. Introduction

High-intensity coherent X-ray free-electron lasers (FELs) have promoted unprecedented advancements in research fields like ultrafast science, sub-nanometre structure characterization and atomic inner-shell electron spectroscopy (Bostedt *et al.*, 2016 ▸; Rossbach *et al.*, 2019 ▸; Li *et al.*, 2022 ▸). Unlike conventional optical lasers, FELs utilize the high-quality electron bunches as gain media to achieve flexible coherent amplification of radiation with spectral coverage down to atomic level withstanding the destruction of strong pulses. In most existing FEL facilities, the accelerated bunches directly pass through the undulator arrays to conduct the high-gain process with the self-amplified spontaneous emission (SASE) principle (Emma *et al.*, 2010 ▸; Ackermann *et al.*, 2007 ▸; Ishikawa *et al.*, 2012 ▸). The relative bandwidth of the SASE-FEL pulses is of the order of the dimensionless Pierce parameter ρ (Bonifacio *et al.*, 1984 ▸), typically ranging from 10^−4^ to 10^−3^.

However, there is strong interest in generating ultrashort and large-bandwidth (10^−2^) FEL pulses, which have potential applications in femtosecond crystallography (Chapman *et al.*, 2011 ▸; Schlichting, 2015 ▸; Nass *et al.*, 2021 ▸), X-ray absorption fine-structure spectroscopy (Popmintchev *et al.*, 2018 ▸) and multi-wavelength anomalous diffraction (Favre-Nicolin *et al.*, 2012 ▸; Son *et al.*, 2011 ▸). In order to broaden the bandwidth of the radiation, various methods have been developed in the last decade. According to the resonance condition of undulators,

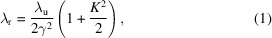

where λ_r_ is the radiation wavelength, λ_u_ the undulator period, γ the Lorentz factor of electrons and *K* the undulator parameter, there are basically two paths to generate large-bandwidth radiation with currently available undulators of fixed period. A possible method is to introduce a transverse tilt in the electron bunch passing through downstream undulators with considerable transverse variation of the undulator parameter like transverse gradient undulators (Prat *et al.*, 2016 ▸) or natural gradient of planar undulators (Song *et al.*, 2018 ▸). This kind of transversely tilted bunches can be achieved by radiofrequency (RF) deflecting cavity or corrugated structure. However, these methods suffer from complicated electron dynamics, dedicated large equipment and enormous cost. A more general method is to increase the energy chirp of the electron beam. Large chirped electron beam can be produced by operating the linac off-crest, and opportunely tuning the bunch compression process in the accelerator. The bandwidth of the radiation is proportional to the energy chirp (Hernandez *et al.*, 2016 ▸; Yan & Deng, 2019 ▸; Prat *et al.*, 2020 ▸). With the energy-chirped electron bunches, the bandwidth can be further increased through the longitudinal space charge (LSC) effect resulting from the extremely compressed electron bunch (Serkez *et al.*, 2013 ▸). However, increasing the energy chirp exploiting the RF is inefficient, since in this case we have a limitation of the RF amplitude and frequency. Furthermore, this would have an impact also on other beamlines in FEL complexes that do not have dedicated linacs to separately tune the beam energy.

On the contrary, the wavelengths of optical lasers are much shorter than that of RF waves, which are usually used to introduce the energy chirp for more efficient electron beam manipulation. It has been proposed that 13 m-long corrugated structure sections can be used to broaden the SASE bandwidth by up to 3% at the European XFEL (Zagorodnov *et al.*, 2016 ▸). Recently a new method based on plasma wakefield acceleration to generate large-bandwidth XFEL radiation has been proposed (Peng *et al.*, 2023 ▸). However, for these approaches a reliable implementation is still a critical aspect, due to the large cost and the synchronization. Thus, passive and dephasingless terahertz wakefield components, dielectric-lined waveguides (DLWs), are employed to efficiently and stably manipulate the bunch with a Cherenkov-style self-wakefield. When the beam passes through the DLWs, the THz longitudinal electromagnetic field can be excited with a gradient order of GW m^−1^ for nC bunches (O’Shea *et al.*, 2014 ▸, 2016 ▸). Besides, the wakefield elements find their applications in phase space linearization (Craievich, 2010 ▸), dechirping operation (Antipov *et al.*, 2014 ▸; Emma *et al.*, 2014 ▸), two-color FEL schemes (Bettoni *et al.*, 2016*a* ▸, 2021 ▸), terahertz sources (Cook *et al.*, 2009 ▸; O’Shea *et al.*, 2019 ▸; Floettmann *et al.*, 2021 ▸), current shaping (Andonian *et al.*, 2017 ▸), beam temporal profile measurement (Bettoni *et al.*, 2016*b* ▸) and efficient particle acceleration (Jiang *et al.*, 2012 ▸).

In this paper, a simple and cost-effective method is proposed to generate a spectral-coherent large-bandwidth X-ray FEL with cylindrical DLW structures. In the proposed method, a DLW structure is employed to generate stable energy chirp in the electron beam taking advantage of the passive, dephasingless and efficient wakefields. The large energy chirp will result in large-bandwidth radiation in the downstream undulators. Three-dimensional start-to-end simulations with the parameters of the Shanghai Soft X-ray FEL facility (Zhao *et al.*, 2017 ▸) have been carried out and the results show that tunable large-bandwidth radiation can be achieved with a bandwidth ranging from 1.01% to 2.16% full width at half-maximum (FWHM) by changing the taper profile of the undulator section.

## 2. Layout and analytic method

**Figure 1:**
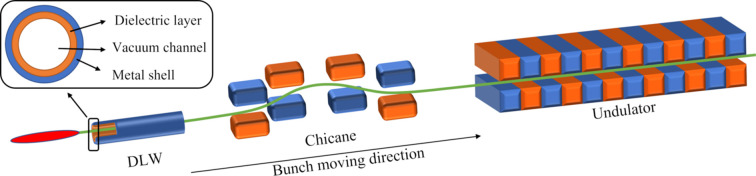
Schematic layout of the proposed scheme (not to scale). Bunches produced in an upstream linac pass through the DLW, chicane and the undulator section successively from left to right. The upper-left subfigure shows a cross-sectional view of the DLW structure, where the dielectric layer in this study is fused silica.

In this method, all the phase space manipulations of the bunch are carried out downstream of the switchyard, to avoid the deteriorative impact on other beamlines. A schematic layout of the proposed method is shown in Fig. 1 ▸, where a cylindrical dielectric-lined metal waveguide has been employed after the linac to induce large energy chirp in the electron beam. Compared with a corrugated structure, the quadrupole field of the DLW is relatively weak while exciting strong longitudinal wakefield. The high-quality flat-top electron bunch generated in the upstream linac is transferred through the 100 mm-long DLW tube made of fused silica (ɛ_r_ = 3.75 as an example) along the axis. Due to the induced resonant Cherenkov-style wakefield excited by the bunch itself, energy modulation is imprinted mainly by the longitudinal electric field. The resonant frequency can be well tuned with different dielectric plating parameters. Wakefield excited can be enhanced as the bunch size decreases in both transverse and longitudinal direction, allowing for adoption of thinner waveguides and therefore shorter modulation wavelengths and stronger amplitudes. From the cosine essence of the point-charge wakefield, the chirp of the bunch head is negative, which means the particle energy of the front slices is higher than that of the back slices. Positive energy chirp can be generated by decreasing the diameter of the waveguide to shorten the wavelength of the wakefield excited, generally. With a following chicane, the positively chirped bunch can be compressed to tune the duration. And an isolated current spike can be obtained with a peak current of several kA and pulse duration of the few femtoseconds level after extreme compression in the downstream chicane. An ultrashort radiation pulse with tunable large-bandwidth can be therefore expected in the following undulator sections.

The wakefield is excited following charged particles passing by dielectric materials. To further collect and enhance this radiation, cylindrical dielectric-lined waveguide structures are utilized. The particles are meanwhile modulated by the field. In order to calculate the wakefield, the steady-state wakefield solution of Maxwell’s equations is used to numerically calculate the bunch evolution in the waveguide. This method is verified efficient when the length of the DLW is much larger than the wavelength of the waveguide’s fundamental wavelength in the following content. The general solution for the longitudinal wakefield potential of the monopole resonant mode along the axis of the dielectric tube is shown as (Ng, 1990 ▸; Rosing & Gai, 1990 ▸) 

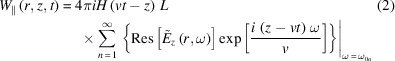

where *z* is the axial coordinate along the tube, *v* is the velocity of the particle, ω_0*n*_ is the *n*th resonant angular frequency, *H*(*vt* − *z*) is the Heaviside function, and *L* is the length of the DLW. The amplitude function 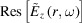 of the vacuum area can be numerically calculated by solving the boundary problem of Maxwell’s equation in the case of cylindrical coordinates. The energy modulation Δ*E*(*s*) introduced within a bunch can be obtained through a convolution of the longitudinal electric field (point charge wake) with the longitudinal bunch distribution.

## 3. Simulation setups

**Figure 2:**
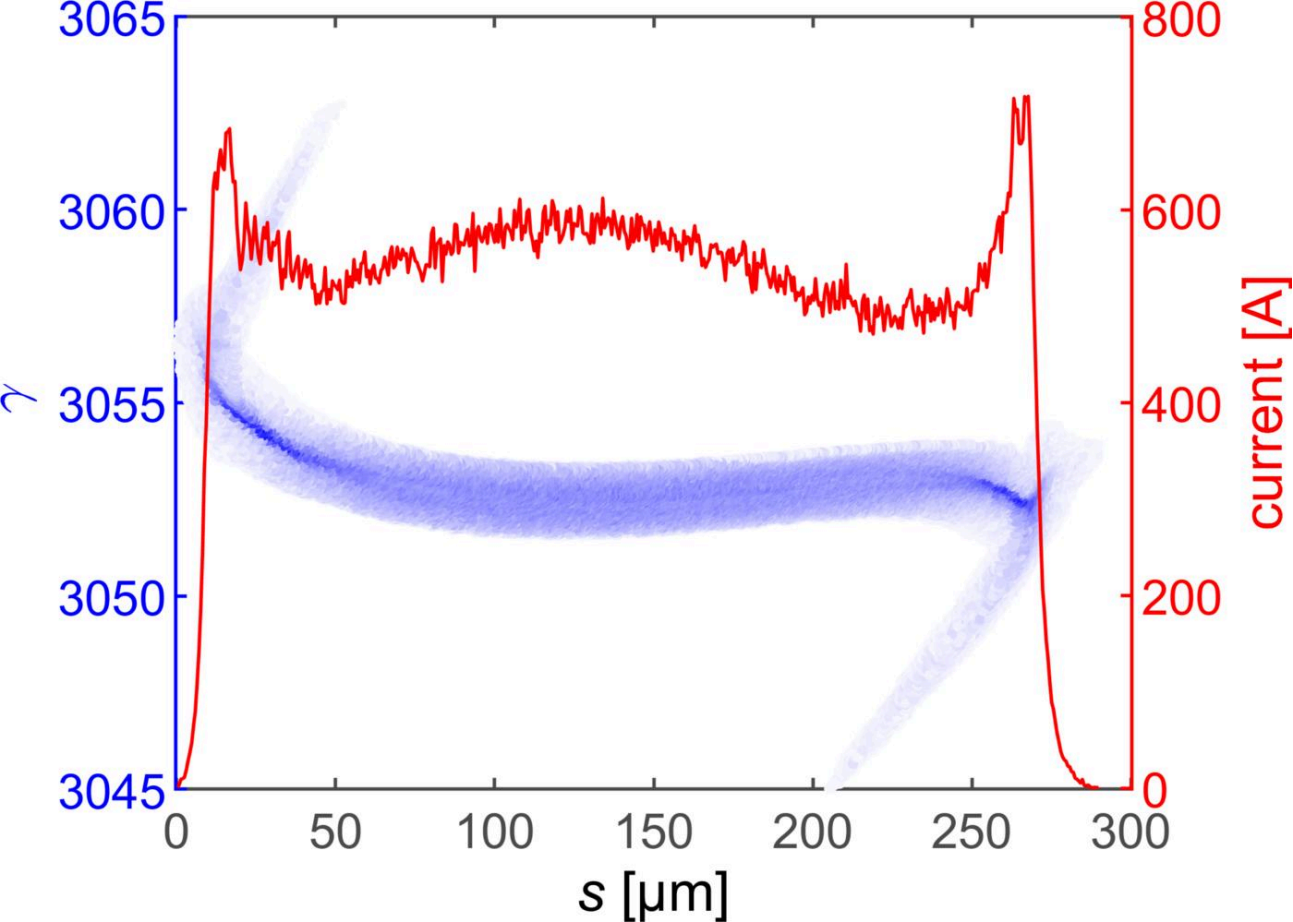
Longitudinal phase space and current profile of the flat-top electron bunch at the entrance of the DLW. The bunch head is on the right.

In order to demonstrate the performance of the proposed scheme, the wakefield simulation code *ECHO2D* (Zagorodnov & Weiland, 2005 ▸) and 3D tracking code *GENESIS* (Reiche, 1999 ▸) are used to verify the bunch dynamics in DLW and simulate the radiation process in the undulators with the typical parameters of the Shanghai Soft X-ray FEL facility (Liu *et al.*, 2021 ▸), as listed in Table 1 ▸. The coherent synchrotron radiation (CSR) effect during compression, the resistive wall wakefield and the LSC effect after compression are also evaluated. The longitudinal phase space and current profile of the electron bunch at the entrance of the DLW are given in Fig. 2 ▸.

## 4. Wakefield and beam dynamics

**Figure 3:**
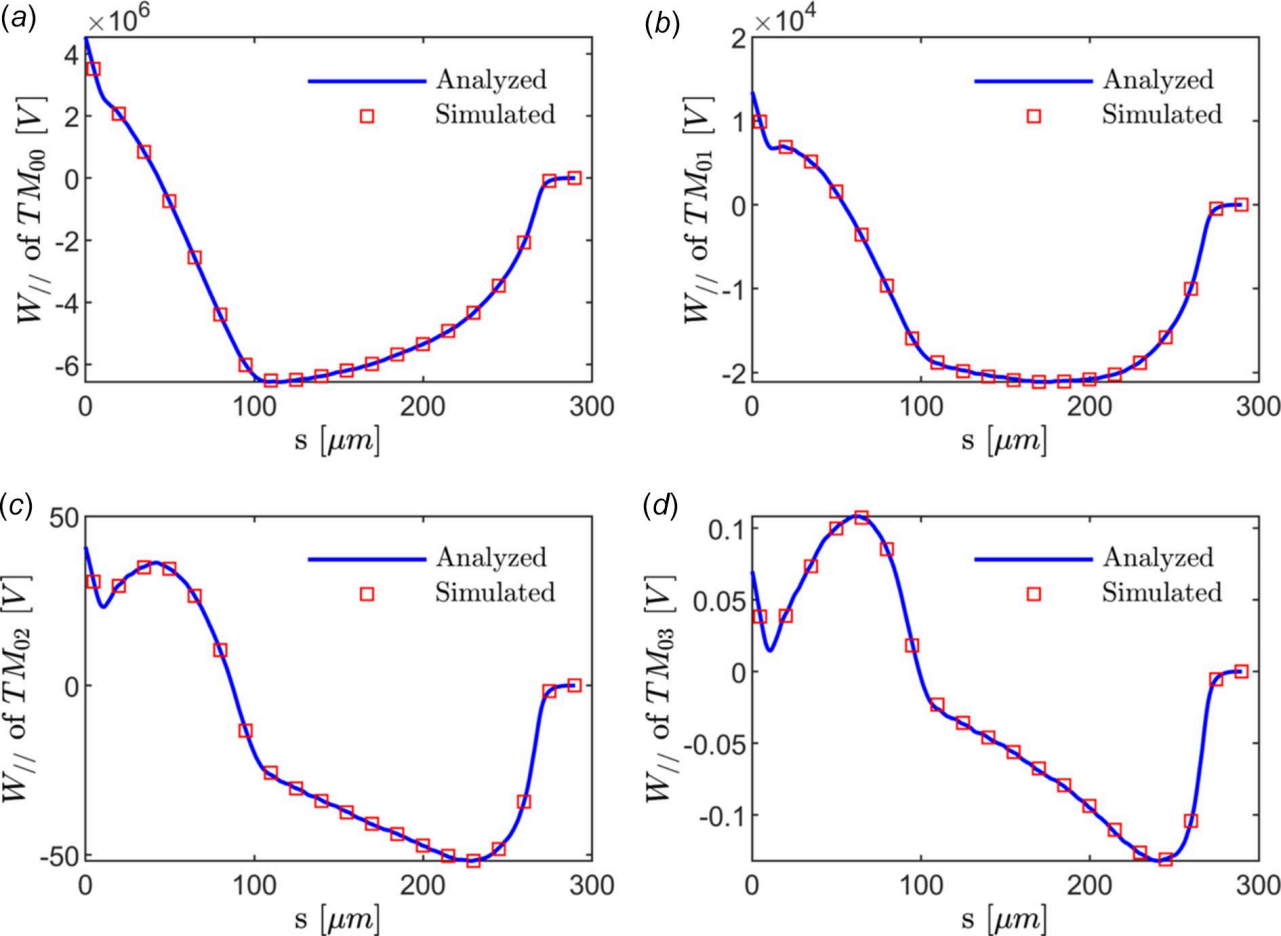
Comparison of the longitudinal wake potential at the exit of the DLW from analytical results (blue solid line) and dynamic simulation results from *ECHO2D* (red squares) for a monopole (*a*), dipole (*b*), quadrupole (*c*) and hexapole (*d*) field under the same conditions. The longitudinal coordinate *s* is defined with respect to the bunch tail. The calculated wake potential sums the first 15 resonant frequencies for each mode. The transient effect at the entrance and exit can be reasonably excluded.

The high-quality electron beam generated in the upstream linac is transported through the 100 mm-long DLW structure on-axis. The theoretical analysis of the steady-state solution with equation (2) and evolutionary simulation results using *ECHO2D* of longitudinal wakefields of the first four modes are summarized in Fig. 3 ▸, from which one can find that the theoretical results agree very well with the simulation case. It is reasonable to simulate the field evolution analytically without considering the transient effect at the entrance and exit. Furthermore, it is evident that the longitudinal wakefield potential of the monopole significantly influences the bunch compared with the higher-order modes.

The first four resonant frequencies of the monopole field, also known as the transverse magnetic (TM) mode, are analyzed to be 0.64, 2.09, 3.78 and 5.53 THz. With the phase velocity of the resonant frequencies equal to that of ultra-relativistic electrons, the fundamental-mode wavelength of the wakefield is estimated to be 0.46 mm, assuring sub-cycle modulation along the electron bunch. According to the results shown in Fig. 3 ▸(*a*), an ∼100 GV m^−1^ quasi-linear energy chirp can be introduced by the DLW structure to the last third of the bunch.

**Figure 4:**
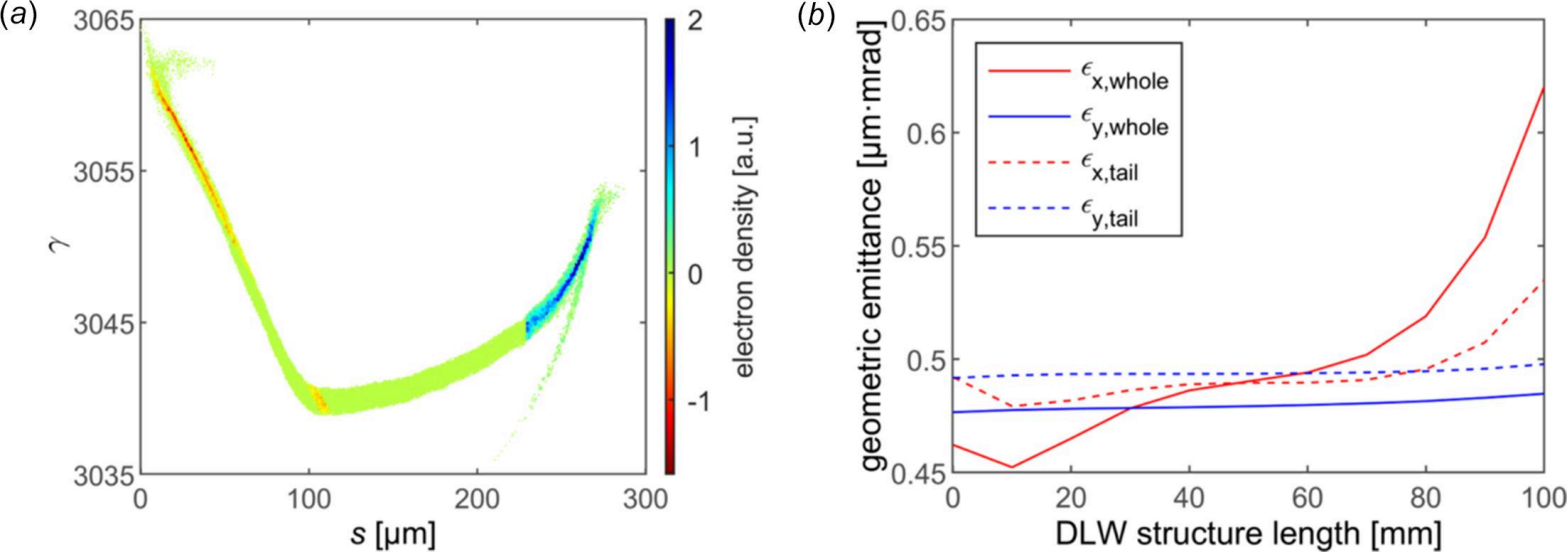
The bunch evolution during wakefield modulation. (*a*) Longitudinal phase space distribution at the exit of the DLW. The negative electron density represents a target compressed section with positive chirp. (*b*) Projected geometric emittance evolution of the whole bunch (solid line) and the positively chirped part (dashed line) in the *x* (red) and *y* (blue) direction in the DLW.

In the modulation process, the time-dependent dipole and quadrupole field arising from off-axis particles would significantly degrade the quality of the electron bunch. To suppress the impact of a transverse wakefield, the electron bunch should pass through the DLW structure along the axis with a nearly round cross section as slim as possible. More precisely, even under this circumstance of conceivable alignment of the whole bunch, the off-axis particles and fluctuations of the slice centroid still make a difference to some degree. However, the fluctuation tends to be suppressed with smaller transverse size. Thus, the Twiss parameters at the entrance of the DLW structure are taken to be β_*x*_ = β_*y*_ = 2 m and α_*x*_ = α_*y*_ = 1, namely about 30 µm transverse beam size to diminish the time-dependent transverse phase space rotation. The longitudinal phase space after modulation is shown in Fig. 4 ▸(*a*). The monopole dominates the longitudinal phase space with such small bunch size. The energy chirp in the last third is estimated to be 103 GV m^−1^. Suffering from the transverse wakefield, the trajectory offset and phase space rotation lead to significant projected emittance growth in the *x* direction from 0.46 µm mrad to 0.62 µm mrad, as shown in Fig. 4 ▸(*b*). However, the quality of the core part with positive energy chirp is preserved relatively better.

In the modulation process, about 0.33% particle loss events take place at the entrance of the waveguide. Those particles with large betatron oscillation amplitude can be intercepted by collimators upstream. In addition, almost no particle hits the dielectric layer in the modulation process, thus avoiding issues like charging and particle shower. As shown in Fig. 3 ▸(*a*), the wakefield is about 60 MV m^−1^, which is far below that of the breakdown threshold (Thompson *et al.*, 2008 ▸). The total energy variation of the bunch before and after the DLW is 58.61 nJ, well within the capabilities of cryocoolers.

## 5. Spectral-coherent large-bandwidth lasing

Large-bandwidth lasing can be obtained when directly guiding the modulated chirped bunch to the downstream undulator sections, as for from the resonance condition. From the differentiation of equation (1), a chirped bunch with uniform current profile would generate radiation with a relative bandwidth twice the value of the relative energy spread. For a chirped bunch with a relative energy spread of 0.65% (FWHM), it is estimated that radiation pulses with a relative bandwidth of about 1.3% can be produced in the undulator section. However, the spectra of such pulses directly generated are incoherent with many longitudinal modes, for there is no external seed laser nor phase coupling between different longitudinal modes.

**Figure 5:**
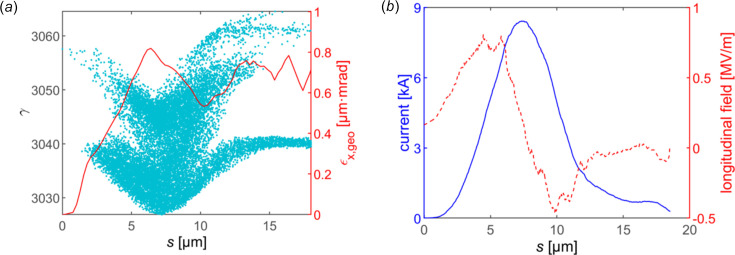
Property of the compressed current spike at the exit of the chicane. (*a*) Longitudinal phase space distribution and slice emittance profile in the bending plane influenced by CSR. (*b*) Current distribution and the longitudinal field of the extremely compressed current spike. The longitudinal field is composed of both the longitudinal space charge effect and longitudinal wakefield of the vacuum aluminium round beam pipe. The longitudinal field induced by the bunch head is reasonably omitted.

To further optimize the phase relation between different frequencies, ultrashort pulses with mode number even down to 1 is expected. The positively chirped part of the bunch is compressed to convert the energy modulation to density modulation in the downstream magnetic chicane with chicane matrix element *R*_56_ of 14.82 mm and total length of 11.8 m so as to promote spectral coherence. This dispersion section is conventional in FEL facilities with external seeding schemes. As a consequence, an isolated current spike with peak current of about 8.5 kA can be generated, which may have a strong influence on the quality of the bunch due to CSR. The code *CSRtrack* (Dohlus & Limberg, 2007 ▸) is adopted to assess the CSR effect in the compression process. By reducing the transverse bunch size in the last dipole, the emittance growth of the current spike can be limited to a certain range (Stulle *et al.*, 2007 ▸). The longitudinal phase space and the horizontal slice geometrical emittance of the current spike at the exit of the chicane are presented in Fig. 5 ▸(*a*). The horizontal slice emittance of the compressed electron beam increases from 0.53 µm mrad to 0.73 µm mrad after passing through the chicane. Besides, the peak current of the compressed bunch reaches up to 8.41 kA while the spike FWHM duration shortened to 5.84 µm, which is already on the scale of the coherence length of 4 nm free-electron lasing, as displayed in Fig. 5 ▸(*b*).

**Figure 6:**
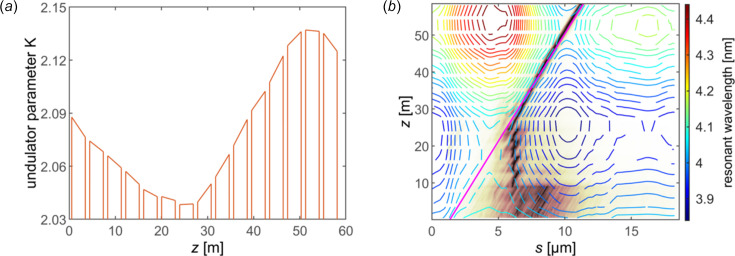
Taper scheme adopted to maintain the resonance condition of a chosen radiation slice. (*a*) The tapered undulator parameter distribution calculated according to the enhanced chirp resulting from the longitudinal field. The profile is the linearly fitted result of each undulator calculated according to the resonant condition. (*b*) Distribution of the resonant wavelength calculated (contour map) along the bunch and the undulator position in the lasing process. The trajectory of the chosen radiation slice is represented by the solid magenta line. The normalized power evolution of a typical ultrashort pulse lasing process is also displayed, of which the peak finally overlaps with the chosen radiation slice.

The compressed electron bunch is transported into the downstream undulator sections after alignment to further study the radiation property after an 8.8 m-long matching section. For such an ultrashort current spike with high current, the bunch will continuously undergo space charge effects from the bunch itself and resistive wall wakefield excitation while passing by aluminium round beam pipes even in the drift section. The longitudinal field comprising the LSC (Ding *et al.*, 2009 ▸) and resistive wall wakefield is calculated and shown in Fig. 5 ▸(*b*). The total longitudinal field can reach up to 0.8 MV m^−1^. Even in the worst case of applying all the longitudinal modulation at the entrance of the 58.5 m-long undulator sections, this would result in longitudinal particle slippage of no more than 0.59 µm with respect to the initial current peak, about an order of magnitude smaller than the current spike width. Thus, it is reasonable in simulation to freeze the longitudinal field and current distribution in this study regarding the numerical noise. The simulation process including these longitudinal effects is carried out using *GENESIS*. To preserve the resonant condition for the target lasing slice with the increasing chirp proportional to undulator distance, the undulator parameter profile should be tuned according to the varying central energy met by the chosen radiation slice along the electron beam, as displayed in Fig. 6 ▸(*a*). In Fig. 6 ▸(*b*), the resonant wavelength distribution for different slices at different undulator distance is shown, with the magenta solid line representing the trajectory of the chosen radiation slice. The radiation peak finally coincidences with the slice. As a result of the increasing chirp generated by the longitudinal field, the pulse duration decreases quickly.

**Figure 7:**
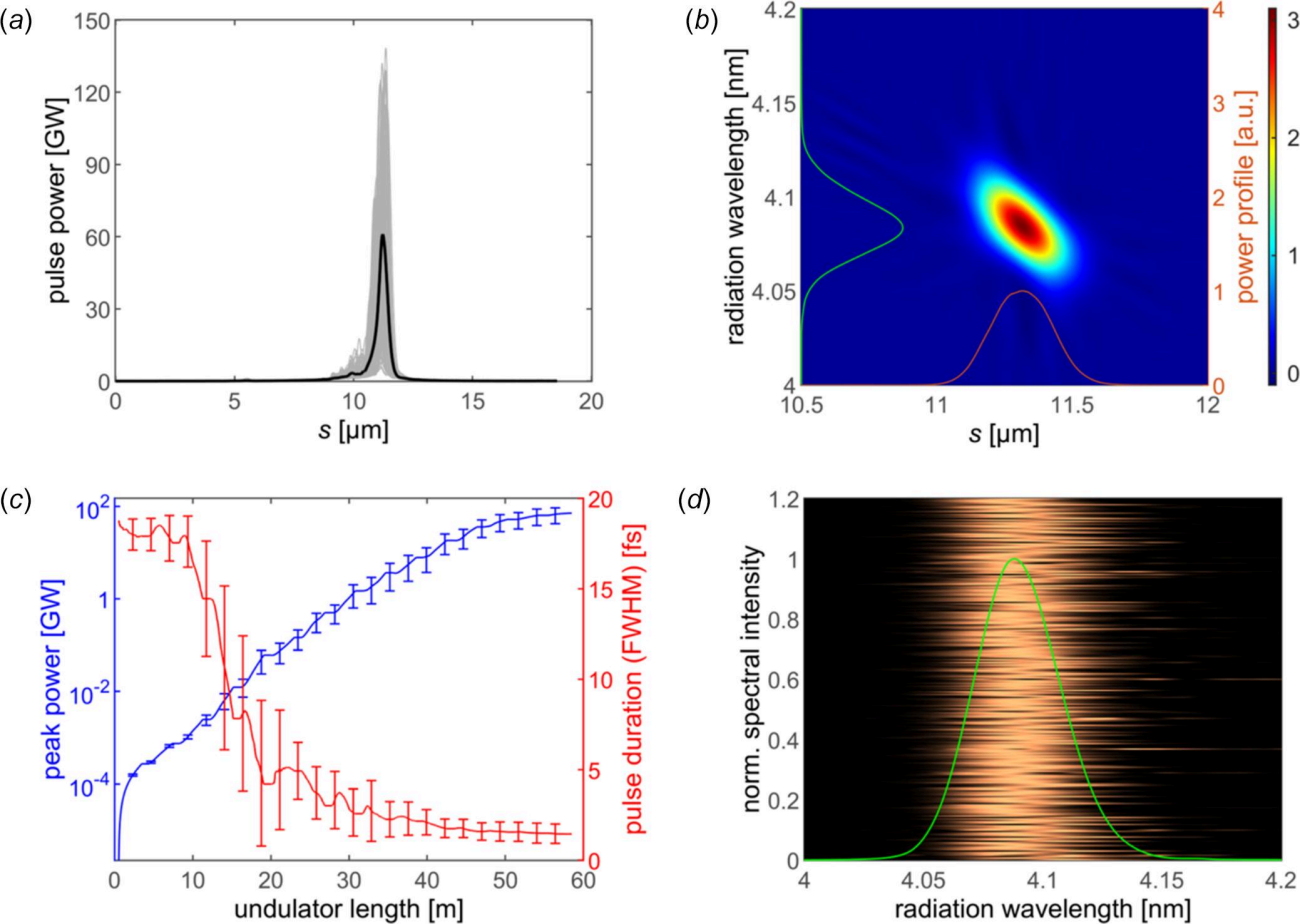
(*a*) The average pulse power profile (black line) and final power profiles distribution of 260 shots (gray line). (*b*) The farfield Wigner distribution for a typical pulse. The normalized power (red solid line) and spectrum intensity (green solid line) distribution are also displayed. (*c*) The statistical results for evolution of mean peak power (blue line) and FWHM duration (red line) of 260 pulses shots along the undulator beamline. (*d*) Average farfield spectral intensity (green line) of the 260 pulses (background image) at the exit of the undulator beamline.

The FEL performance has been summarized in Fig. 7 ▸. As a result of the large energy chirp induced by the longitudinal field and the matching undulator taper profile, it can be found in Fig. 7 ▸(*a*) that isolated X-ray pulses without sidebands are stably produced with different initial shot noises. The average pulse energy is about 131 µJ. The Wigner distribution of a typical shot is shown in Fig. 7 ▸(*b*), in which the bandwidth of the spectral intensity is about 0.77%. The positive chirp can be attributed to the chirp of the resonant wavelength around *z* = 40 m in Fig. 6 ▸(*b*). The statistical FEL evolution process for 260 shots is summarized in Fig. 7 ▸(*c*) where one can find that the average FWHM pulse duration decreases from 19 fs to about 1.41 fs along the 15 undulator segments resulting from increasingly enhanced energy chirp, and the peak power finally increases to 71 GW. As depicted in Fig. 7 ▸(*d*), the average central wavelength for 260 shots is around 4.08 nm with a relative bandwidth of 1.02% (FWHM). It should be pointed out that only the current spike of the beam is taken into account in the FEL simulations, because the gain process of the head part is eroded compared with the target region due to the low current, transverse mismatch and off-resonant undulator parameters. The power of the head part is lower by a factor of 100000 than the peak power in further simulations. In addition, the betatron oscillation due to mismatch is limited to 0.5 mm. Therefore the particle loss in the undulator sections is controllable.

**Figure 8:**
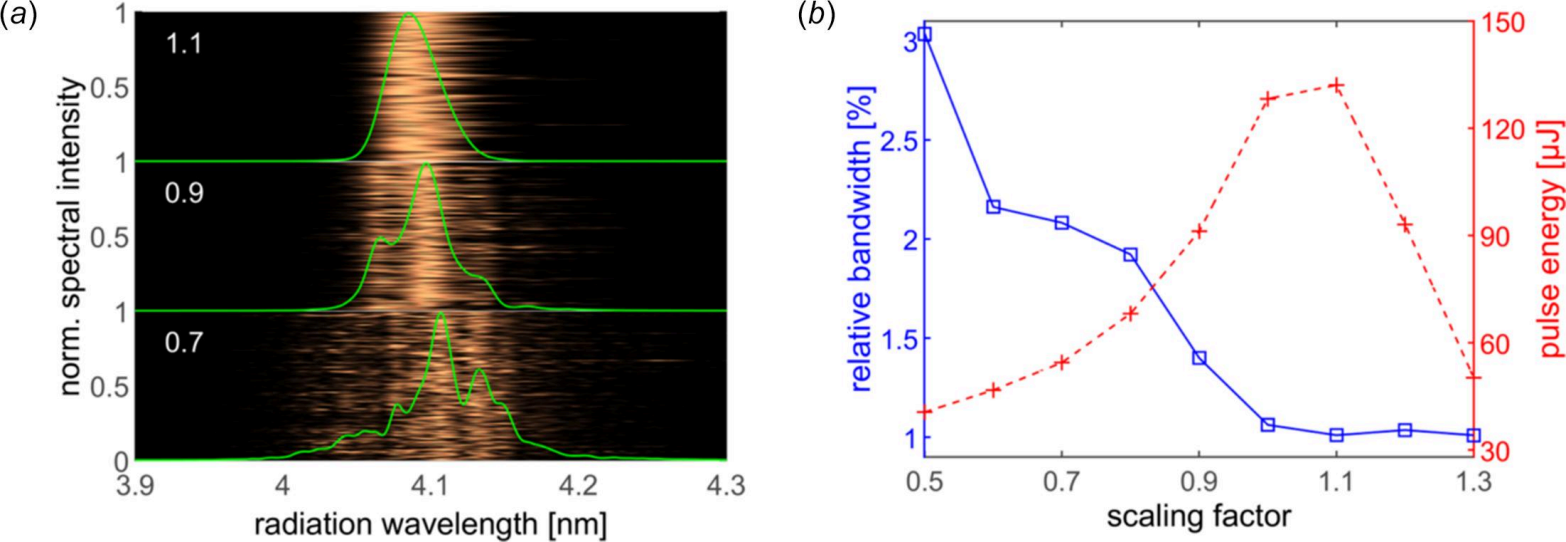
(*a*) Normalized spectra accumulated for 180 shots with a scaling factor of 1.1, 0.9 and 0.7. (*b*) The average bandwidth (blue solid line) and pulse energy (red dashed line) for 180 shots as a function of the scaling factor.

In the foregoing simulation, the undulator taper profile is chosen to preserve the resonant wavelength as the target slice slipping ahead, namely the ‘chirp-taper’ technique (Saldin *et al.*, 2006 ▸; Giannessi *et al.*, 2011 ▸). Taking advantage of the high current spike, the bandwidth can be further broadened by disturbing the resonance condition. Fig. 8 ▸(*a*) displays the radiation property at the exit of 15 undulator sections of different scaling factor. The scaling factor is defined as the taper strength with respect to the optimized one shown in Fig. 6 ▸(*a*), characterized as *K*_*n*_ = *n*(*K*_*n*=1_ − *K*_0_) + *K*_0_, where *K*_*n*_ is the undulator profile for scaling factor *n*, *K*_0_ is the initial undulator parameter, and *K*_*n*=1_ is the resonant undulator profile as demonstrated in Fig. 6 ▸(*b*). In this manner, the bandwidth can be tuned from 1.01% to 2.16% (FWHM) by adjusting the scaling factor from 0.5 to 1.3 as summarized in Fig. 8 ▸(*b*). Meanwhile, the total pulse energy decreases by a factor of three from 134 µJ to 46 µJ when the bandwidth is extended by a factor of near two. Further enhancing the taper scaling factor, the final radiation property degrades sharply as a consequence of the short growth length preserving the resonance condition.

## 6. Conclusion

In this paper, a novel method is proposed using DLW to induce a large positive energy chirp in the electron beam without interfering with the running of the linac and other beamlines in a FEL complex. By use of a 100 mm-long DLW tube, stable sub-cycle energy modulation can be achieved taking advantage of the passiveness and dephasingless wakefield of the DLW structures. Three-dimensional simulations have been carried out and the results show that the isolated ultrashort X-ray radiation pulse can be achieved with a FWHM bandwidth of 1.02% and peak power of 71.14 GW. By rescaling the taper strength of the undulator segment the bandwidth can be further tuned from 1.01% to 2.16% (FWHM). The longitudinal space charge effect, the coherent synchrotron radiation effect and the longitudinal resistive wall wakefield are taken into account. The isolated large-bandwidth X-ray radiation pulses will have great potential applications on spectroscopy science studies.

## Figures and Tables

**Table 1:** Main parameters used for simulations

	Parameter	Value	Unit
Bunch	Average energy	1.52	GeV
	Slice energy spread	0.46	MeV
	Normalized emittance	1.43	mm mrad
	Total charge	500	pC
	Bunch length (FWHM)	0.26	mm

DLW	Relative permittivity	3.75	
	Inner diameter	0.4	mm
	Outer diameter	0.5	mm
	Waveguide length	100	mm

Chicane	*R*_56_	14.82	mm

Undulator	Undulator parameter, *K*	2.08	
	Period, λ_u_	23.5	mm
	Undulator length	3	m
